# Science based public policies: Lessons from Covid19 on the use of randomized trials

**DOI:** 10.1590/1678-4685-GMB-2020-0273

**Published:** 2021-01-20

**Authors:** Natalia Pasternak Taschner, Carlos Orsi

**Affiliations:** 1 Instituto Questão de Ciência, São Paulo, SP, Brazil.; 2 Universidade de São Paulo, Instituto de Ciências Biomédicas, SP, Brazil.

**Keywords:** Randomized trials, scientific method, equipoise, social sciences

## Abstract

The current SARS-CoV-2 pandemic gave rise to a spirit of methodological anarchy in some fronts of biomedical research, embraced by some under the excuses of urgency and time restraints. This movement, however, comes at the same time when social sciences begin to recognize the value and soundness of the clinical research rationale - the need for randomization, of fair comparisons between intervention groups, the humility of acknowledging ignorance and accepting uncertainty, these last two imperatives usually subsumed under the principle of “equipoise”.

## Introduction

Randomized controlled trials (RCT) have been challenged during Covid-19 Pandemic (Zanotto, 2020)⁠. Although known as the most reliable degree of evidence to evaluate a medical intervention, many have questioned their true validity, claiming that there was no time to properly run them, due to the global emergency, and that observational studies were just as good, with the advantage of being much faster and cheaper to run (Gautret *et al.* 2020)⁠. More than the sense of urgency, the true need for RCTs was questioned, as if they were a waste of time and money, an exquisite luxury, and unnecessary: the pandemic becomes an excuse for a methodological free-for-all (Alexander *et al.* 2020)⁠. The scientific community did not see that coming. We certainly did not expect that after almost 200 years from the first randomized trial, and the way that this kind of experiment changed the course of health and medicine, we would have to explain - again - why randomized trials are the best way to determine cause and effect. And why correlation is not causation. 

## A bit of history

The first randomized trial in history is believed to have happened in England, in 1753 (Milne, 2012)⁠⁠. James Lind was a navy doctor, in charge of the crew of the “Salisbury”. At that time, scurvy killed more sailors than the war. Lind was skeptic of the regular medicine of the time, which was based mainly on bloodletting practices. Having read old navy diaries, he noticed that vessels carrying fruit had fewer cases of scurvy among the crew. He decided to run what he called “a fair test”. He allocated 12 sailors in pairs, “as similar as I could have them”. He took care that each pair received exactly the same treatment, but for one intervention: vitriol, cyder, vinegar, lemons and oranges, sea water and a mixture if garlic, mustard seed and radish root. Lind’s success in demonstrating that citrus fruit cured scurvy not only saved lives but also inspired others to think critically and adopt the “fair test” idea. 

The ideia that the best way to establish cause and effect relationships is to compare very similar groups or situations that, however, obtain divergent endpoints - the “cause” of the divergence being one (ideally, the only one) of the significant differences between the groups or situations - wasn’t new then. Galileo Galilei used it in his book “The Assayer”, published in 1623. Later, in 1843, philosopher John Stuart Mill would systematize this “method of differences” in his book “System of Logic”.

More than fifty years after Lind, but still more than a decade before Mill, in 1816, Alexander Hamilton, a military surgeon serving in Portugal, describes another randomized trial in his MD thesis (Milne and Chalmers, 2015)⁠⁠. Patients were randomized to be treated by three doctors, only one of whom used bloodletting. The number of deaths was ten times higher in this group. The idea of a “fair test”, consisting mainly on dividing subjects into similar groups to test an intervention was gradually refined over time, until reaching its full “randomized, double-bind placebo-controlled trial” format considered today to be the gold standard in clinical trials. Randomized trials became increasingly popular after Archie Cochrane, considered to be the father of evidence based medicine wrote on his book “Effectiveness and efficiency” that “You should randomize till it hurts” (Shah and Chung, 2009)⁠⁠. 

RCTs brought us validation of vaccines, medications and every kind of medical intervention in modern medicine. And it proved such a valuable tool that it has recently made a debut in other fields. After all, a “fair test” can be used in any field, to demonstrate true cause and effect: Galileo and Mill, for instance, weren’t talking about Medicine specifically when they wrote about comparisons and differences (Drake, 1957; Lipton, 2004). Observational studies - where we try to abstract cause and effect relationships from mere sequences of events, or from comparisons between groups we don’t know to be properly comparable - can be misleading due to confounding factors and biases. The impact of this line of thought in Medicine has been enormous: bloodletting is described in one of the oldest medical texts known, the Egyptian Ebers papyrus from circa 1550 BCE (Parapia, 2008)⁠⁠, and was standard medical practice in the Ancient World, during the Middle Ages and right into the Modern Era. It took thousands of years and controlled trials for its harm to be dully recognized. It is now dully accepted that in medical interventions, doctor and patients are subject to confirmation bias and placebo effects that can compromise results, the same rational thinking hasn’t always been applied to social and economic studies. In fact, one of the first studies in economics to rely on RCT was published in 2003, possibly one of the first steps of the author towards the Nobel Prize in Economics, in 2019 (Kremer, 2003)⁠. 

These studies are also prone to human bias, and confounding factors. How do you determine the true cause of lower rates of vaccination, or poor school achievement in developing countries? The old precept “give a man a fish, he will eat today, but teach a man to fish and he will eat forever” has been used by distinct political ideologies: while left-wing politicians argue that a man cannot be taught anything on an empty stomach, most right-wing politicians seem to take the quote literally. But the question remains: is it true? has it been tested? And CAN it be tested? 

## Two case studies: India and Kenya

The answer is yes, and using randomized trials to answer social and economic questions has ultimately earned three researchers the Nobel Prize in Economics in 2019 (https://www.nobelprize.org/prizes/economic-sciences/). Among many issues tackled by the team with this scientific approach, we selected two that strongly support the use of science based public policies. 

Rural India suffered from an extremely low vaccination rate. Banerjee *et al.* (2010)⁠ decided to approach the problem with a “fair test”. It is often believed that the main cause for low vaccination rates in developing countries is shortness of supply. However, observation showed that low quality services could also be adding to the problem, since healthcare workers were often absent from vaccination clinics. The authors randomized 134 villages into three intervention groups: group A villages had access to a monthly reliable immunization camp, group B had the same, but with an extra incentive like a bag of lentils, and group C was the control group, with access to the regular vaccination facilities. 

**Figure 1 -:**
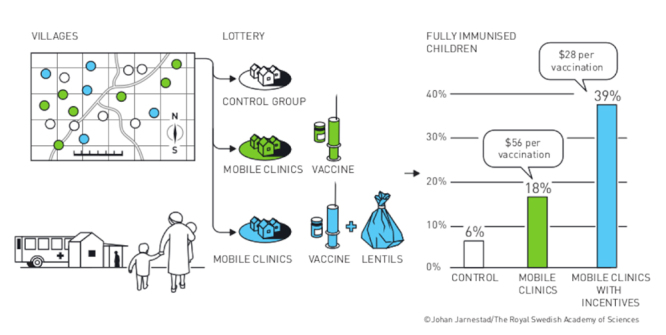
Randomized trial for vaccine uptake in rural India.

The results shown in Figure 1 speak for themselves. Vaccination rates increased the most in the group with access to both availability and incentive. Obvious as it may be, this was never demonstrated to be one of the causes of low vaccination rates. And the best cost-effective solution turns out to be not so obvious: the cost of the bag of lentils was compensated by the number of children vaccinated, making the overall cost drop by almost half the price. This intervention alone did not solve the problem completely, as there are other issues that interfere with vaccine uptake, but it shows a rational way to approach the problem, and it offers a cost-effective partial solution that is very easily implemented. Not to mention how much this intervention is likely to save in public healthcare by preventing infectious diseases in children, a factor not measured by the authors in this trial. 

Another approach made by the team was to apply randomized trials to education studies in Kenya. The interventions are summarized in a review by Banerjee and Duflo (2009)⁠⁠. The authors address how randomized trials have helped to understand that common sense solutions to educational issues, such as hiring more teachers, or offering more textbooks, did not actually work to improve student’s performance, but interventions such as distributing deworming medication was 20 times as effective as hiring a new teacher, and much cheaper. 

Tracking students according to their attainment level and test results - placing them in separate classes selected by prior achievement - has always been frowned upon by educators, who believe it generates inequality and benefits high achieving students only, leaving others behind. Duflo *et al.* (2011) show otherwise. They randomized schools to test this hypothesis and showed that tracking students by prior achievement benefited all students alike. This was counter intuitive and had never been demonstrated clearly in a rigorous test. 

The Nobel Prize winners are not alone in their efforts to apply scientific method to social and economic issues. Jayachandran *et al*. (2017)⁠ randomized 121 villages in Uganda to receive payments for ecosystem services (PES). The ideia was to check if payments would help to contain deforestation. Sixty-one villages were offered PES to conserve their forest. The authors show that tree cover was reduced in both interventions, but only half as much in the PES group. The conclude that the benefit generated by the carbon emission prevented by the program was 2,4 times higher than the cost of the program itself. 

## Equipoise

Taken together, these data strongly suggest that RCTs have a much wider role to play in society than just help us discriminate between medical interventions. However, even for drug and vaccine testing, the ethical basis of the RCTs are often contested - is it right to offer something that may be beneficial to only a portion of the participants of a test? Is it right to allocate people to treatments of conditions that may be harmful? In Medicine, there are ethical research committees to protect trial participants. But just how ethical is it to use randomized tests for public policies? 

In the medical profession, the ethical linchpin of the RCT is the concept of “equipoise”, sometimes defined as “a state of regarding two treatments as an equal bet in prospect” (Edwards *et al.*, 1998)⁠. Ideally, medical equipoise is a state of “perfect ignorance” about the merits of the treatments being tested: it is stipulated that as soon as there’s conviction that one treatment is the best, it becomes unethical to keep part of the patients in an inferior condition.

The demand for “perfect ignorance” may be too strong, however; psychologically, it is hard to see how someone would start a trial without at least a hint that the treatment under scrutiny is better than the standard of care (or than doing nothing). 

To circumvent this objection, some authors have proposed expanding the concept, going from personal to clinical (Freedman, 1987)⁠⁠ or community (Karlawish and Lantos, 1997)⁠⁠ equipoise, defined no longer as a state of ignorance or uncertainty of the individual researcher or research team, but of the medical profession (clinical equipoise) or the public at large (community). De Meulemeester *et al.* (2018)⁠ go to suggest that true equipoise should be assessed vis-avis the pertinent literature.

In the social sciences, the equipoise problem is compounded by the ongoing tension between the “normative” (discussions of what is wrong with society and how it should ideally evolve, usually founded in value-laden concepts like “justice” or “equality”) and “positive” (the search for objective social-science laws, principles and regularities) approaches (Israel, 1972; Goddard, 1973)⁠. Many fields in the social sciences are divided by schools of thought that could conceivably disagree, in principle, on which hypotheses have, or not, legitimate equipoise: a universal basic income policy could be seen as ethically mandatory by some theoreticians and as highly dubious by others, for instance.

The supposed “incommensurability” of research traditions - that different theoretical frameworks speak different languages and may even inhabit different “worlds” - is attributed to philosopher and historian Thomas Kuhn, but he later toned down his assertions on the subject (Schlesinger, 1981)⁠. Other philosophers deny that incommensurability is an insurmountable problem, and note that scientists working on different traditions can, and do agree on the best solution or best explanation for diverse problems or phenomena (Laudan, 1984; Lipton, 2004)⁠. 

Be it as it may, the word “equipoise” doesn’t appear in the “Dictionary of the Social Sciences” published by Oxford University in 2002 (Calhoun, 2002)⁠, and in 2013 the prestigious British Medical Journal published a paper with the title “In Search of Social Equipoise” (Petticrew *et al.*, 2013)⁠ lamenting the lack thereof. “No concept of ‘social equipoise’ exists”, the author points out. “This makes it politically difficult for policy makers to acknowledge uncertainty and to conduct evaluations. The development of ‘social equipoise’ may help foster a greater culture of evaluation outside medicine” (Petticrew *et al.*, 2013)⁠.

According to Petticrew *et al.* (2013)⁠⁠, the filling of this conceptual vacuum will require a “legitimization of uncertainty”. 

The still-ongoing debates on the quality of the clinical science produced during the pandemic shows that such “legitimization” is always under dispute, even in the medical profession. Earlier on, voices were raised against “pandemic research exceptionalism” (London and Kimmelman, 2020)⁠, but went largely unheard. 

Restoring the proper place of equipoise and randomization in the biomedical sciences is a task that lays ahead, and should be done together with the establishment and legitimization of these concepts in the social sciences, or at least in the interface between social sciences and public policy. 

The track record of the 2019 winners of the prize in Economic Sciences in Memory of Alfred Nobel and other initiatives, like the work of Jayachandran *et al.* (2017)⁠ show that putting old ideological certainties on hold and recognizing true uncertainties can bring benefits for those who are most in need.
